# The Angle of Trunk Rotation in School Children: A Study from an Idiopathic Scoliosis Screening. Prevalence and Optimal Age Screening Value

**DOI:** 10.3390/ijerph16183426

**Published:** 2019-09-16

**Authors:** Katarzyna Adamczewska, Marzena Wiernicka, Ewa Malchrowicz-Mośko, Joanna Małecka, Jacek Lewandowski

**Affiliations:** 1 Poznan University of Physical Education, Faculty of Health Sciences, Department of Musculoskeletal Rehabilitation, 61-871 Poznan, Poland; 2 Poznan University of Physical Education, Faculty of Sport Sciences, 61-871 Poznan, Poland

**Keywords:** idiopathic scoliosis, angle of trunk rotation, optimal age screening, prevalence, school children

## Abstract

(1) Background: Idiopathic scoliosis is a deformity of the growing spine. It affects 2–3% of adolescents; yet its cause is still unknown. At the early stage of idiopathic scoliosis (IS), the signs are not very noticeable. That is why the primarily school-based screening for scoliosis is so important. (2) Methods: This was a cross-sectional analysis of 6850 respondents. Participants were elementary school students in the metropolitan area of Poland. The suspicion of IS was based on detection of three-dimensional deformity of the spine using scoliometer. (3) Results: Respondents were divided into two groups: Angle of trunk rotation (ATR) = 0–3º and ATR > 3º. Presented research using a referral criterion of 5º ATR showed that in the group of participants who had ATR > 3º the largest percentage of 5 degree values was recorded at the second and third measurement level of the spine (30.5%, 31.1%, respectively). Analyzing the differences between the two groups of girls (ATR = 0–3º, ATR > 3º), statistically significant differences were recorded between 9 and 11 years of age (*p* = 0.0388). Girls with ATR > 3º at all measuring levels are significantly slimmer than girls with ATR 0–3º; (4) Conclusions: Age; sex, and risk of developing angle of trunk rotation are very closely associated. The main thoracic (level 2) and thoraco-lumbar (level 3) level of measuring of the spine appears to be the most differentiating in the diagnosis of scoliosis. Girls with a lower degree of trunk deformity (4–6º trunk rotation), which can present mild scoliosis and those with a higher degree (7º trunk rotation) have lower body mass than girls within the norm.

## 1. Introduction

Idiopathic scoliosis (IS) is the most common pediatric musculoskeletal disorder that causes a three-dimensional deformity of the spine [1]. It refers to about 2–3% of the adolescent population [2]. Although its etiology is largely unknown, researchers have focused on genetic factors, metabolic and hormonal disorders, and growth asymmetry with mechanical and connective tissue abnormalities. A common diagnostic criterion is a Cobb angle of 10º or more. As is generally known, IS progression appears more frequently among girls than boys, especially during puberty [3]. The optimal age for scoliosis screening is still under debate. The Scoliosis Research Society has recommended screening girls at the age of 11 and 13 years and screening boys at the age 13 or 14 years [4]. Moreover, in recent years, the Scoliosis Research Society and the American Academy of Orthopedic Society of North America, and the American Academy of Pediatrics have endorsed school scoliosis screening programs [5,6]. Researchers from Tokyo suggest that school screening for scoliosis is effective for early detection; however, it is first necessary to review and optimize the target groups [7]. Some countries, like the USA and Hong Kong, report that the school-based screening program is costly and inefficient [2]. The Canadian Task Force on Periodic Health examination, the British Orthopedic Association, and the British Scoliosis Society do not recommend screening [2]. These differences in expert opinion are constantly subject to discussion and research. School screening has generally been performed between ages 10 to 14 years. What is certain, early diagnosis and appropriate therapeutic methods will help to inhibit the progressive changes in the musculoskeletal system [8]. Detection during the initial stage when the deformity is unnoticeable offers an opportunity of non-surgical treatment. That is why scoliosis screening is so important in primarily preventing deformity progression. The basic method for scoliosis detection in school screening programs is examination by using a scoliometer. It has not been designed to be a diagnostic method but to select children with high probability of occurrence of idiopathic scoliosis out of the healthy population [9,10]. The implication the scoliometer measurement of the angle of trunk rotation with combination of the Adam Forward Bending Test has been shown the easiest, non-invasive measure of trunk deformity [11,12]. Scoliometer is an instrument that measures axial trunk rotation in individuals with scoliosis. Frequency analysis revealed relatively good specificity, sensitivity, and predictive capability. Coelho et al. [13] noted the correlation between the scoliometer measurement and the radiographic analysis (r = 0.7 with *p* < 0.05) in their research. The use of this tool as a screening device is appropriate [14,15]. It should be noted that adolescent idiopathic scoliosis (AIS) develop at the age of 11–18 years and most often occurs among IS (comprising 85–90% of cases of IS in children). According to Konieczny et al. [16], AIS occurs in the general population in a wide range from 0.47% to 5%. 

The purpose of this study was to (1) present epidemiological findings according to the age and gender of the participants, (2) to assess the magnitude of trunk asymmetry, (3) find the optimal age screening, (4) and to observe the age of children, especially girls of the highest IS risk, which should not be overlooked in school scoliosis screening. 

## 2. Materials and Methods 

We applied a cross-sectional, observational study. The study was approved by The Poznan University of Medical Sciences Bioethics Committee under process number 892/12. After the previous physiotherapy training, all the methodological guidelines conducted in the study were strictly followed. One month before beginning the data collection, the screeners were trained to use the Bunnell scoliometer.

### 2.1. Sample Size 

The study involved 6850 angle of trunk rotation analyses volunteers (*n* = 6850) who met the inclusion criteria. A total of 65 schools in Poznan (Poland) responded to the study announcement. The inclusion criteria for the examination were the following: Only healthy volunteers between 9 and 13 years of age who took part in the school’s regular physical activity class during the last year were recruited. In addition, the participants had no history of surgery on the back or lower limbs, and a lower limb length discrepancy smaller than 2.5 cm. Parents and legal guardians of all participants signed an informed consent form prior to participation. The volunteers were sought among all the primary schools in Poznan.

### 2.2. Research Procedure

The male volunteers participated in the examination bare-chested. The female volunteers had their hair tied up and wore a customized backless t-shirt to provide full view of their back. After collecting anthropometric measures, examinations of angle of trunk rotation were done using the Bunnell scoliometer. The screening test was quick, easy to perform and repeat, non-invasive, and safe. The examination procedure was carried out in Adam Forward Bending Test position and also allowed to rate the vertebral rotation—the angle of trunk rotation (ATR) [17]. Through palpation, the spinous processes T_1_ to L_5_ and posterior iliac superior spine were located and marked using easy-blend and anti-allergic markers. The measurement was performed in the free standing position during bending (Adams forward test) at three levels: Level 1—proximal thoracic (T_1_–T_4_), level 2—main thoracic (T_5_–T_12_), and level 3—lumbar (T_12_–L_4_) [18,19]. The patients bent their trunk forward until it was parallel to the ground, keeping the palms of their hands together. The volunteers kept their feet approximately perpendicular to the iliac junction line. For all of the sections, an ATR value from zero to 3 was considered the norm, while values above 3 were considered to be outside the norm. The reason of that division is to present the results according to the Bunnell classification percentage of children who are in the range of ATR 4–6 with a 5 degree value. According to Bunnell, these children should be re-examined for scoliosis within 4–12 months, while according to Coelho it is highly probable to diagnose scoliosis in that moment and therapeutic treatment should start earlier [13,20].

Initially, muscle extensor spinae was examined on three levels. Measurements of the angle of trunk rotation were performed once using the qualified assessors. The examination took place in separate bright rooms with adequate thermal conditions during school hours. 

Children with ATR between 4–6 degrees were also strongly suspected in clinical trials for increased trunk asymmetry. Therefore, these volunteers were referred for follow-up examinations within three months.

All statistical analysis was performed using Statistica 8 Software. The data for scoliometric analyses were correlated using Pearson correlation coefficients with a level of significance *p* < 0.0001. This study used independent Z-tests to analyze the average values of measurement items between groups according to age, gender, angle of trunk rotation, and body mass. These groups were used to investigate risk factors of scoliosis, and comparatively analyzed angle of trunk rotation with age, gender, and body mass (ANOVA). We finally checked the distribution of percentage of children who had ATR = 5° in both groups (ATR = 0–3º and >3º) and analyzed the optimal age screening.

## 3. Results

### 3.1. Demographics

Full data were available on 6850 volunteers: 3440 females and 3410 males. The main anthropometric characteristics and the value of trunk asymmetry are described in Table 1.

As shown in Table 2, the division of research material was split into two groups: Participants (girls and boys) with ATR = 0–3º and ATR > 3º. Number of positively screened volunteers was determined based on the ATR >3º criterion. The analysis of trunk asymmetry was analyzed on three levels of the spine: Proximal thoracic, main thoracic, and lumbar. The maximal ATR value was retained.

Among participants who had ATR > 3º, the largest percentage of 5º values was recorded at the second measurement level of the spine (31.5%). A high percentage a five-degree trunk rotation angle was also recorded at level 3 (33.2%) and the lowest at the first level 28.9%, respectively. Therefore, the percentage of five-degree values among the entire population exceeded over 3% in the second measurement level. 

### 3.2. Prevalence

**Figure 1:**
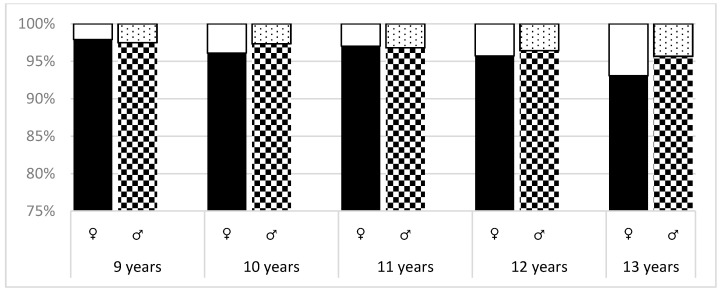
ATR absolute values—comparison of differences between age and sex (level 1). * * In the upper part of the bar are presented children whose ATR is more than 3º, in the lower part ATR = 0–3º (Figure 1, Figure 2 and Figure 3).

As shown in Figure 1, it can be observed that on the first level of measurement, ATR increased in girls with age (*p* < 0.0001). Statistically significant difference (*p* = 0.0829) between ATR values were noted in girls and boys in the 13-year group. Analyzing the differences between the two groups of girls (ATR = 0–3º, ATR > 3º), statistically significant differences were recorded between 9 and 13 years. Girls were noted with a *p*-value of *p* = 0.0006.

**Figure 2:**
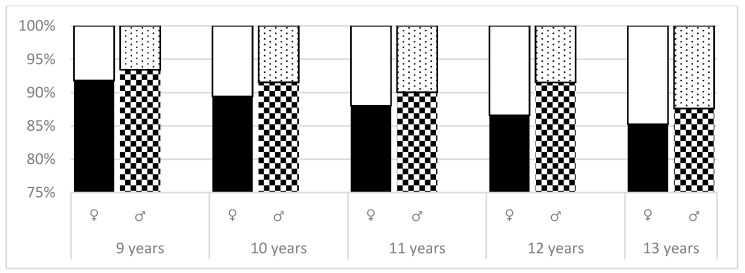
ATR absolute values—comparison of differences between age and sex (level 2).

As shown in Figure 2, at level 2, there was a noticeable increase in percentage of girls who had ATR > 3º in all age groups. Statistically significant differences were found in the group of 12-year girls and boys with *p* = 0.0019. Analyzing the differences between the two groups of girls (ATR = 0–3º, ATR > 3º), statistically significant differences were recorded between 9 and 11 years of age (*p* = 0.0388). The difference between 9 and 13 years girls was noted with a *p*-value of *p* = 0.0023.

**Figure 3:**
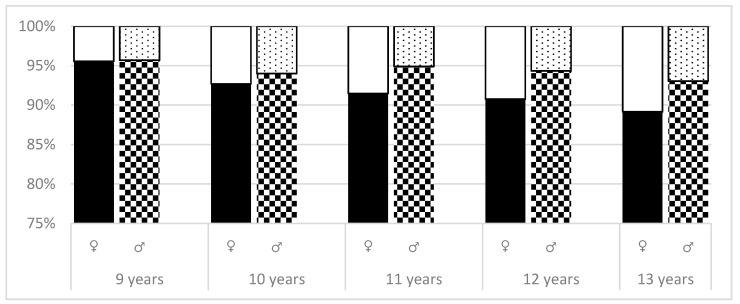
ATR absolute values—comparison of differences between age and sex (level 3).

As shown in Figure 3, the third level of measurement also presented the increase in the percentage of girls who had ATR > 3º in all age groups (*p* < 0.0001). Statistically significant difference (*p* = 0.0050; *p* = 0.0073) between ATR values were noted in girls and boys in the 11 and 12-year group. Analyzing the differences between the two groups of girls (ATR = 0–3º, ATR > 3º), statistically significant differences were recorded between 9 and 11 years of age (*p* = 0.0388). The difference between 9 and 13 years girls was noted with a *p*-value of *p* = 0.0023.

### 3.3. The Difference and Comparison in ATR Values Between Age and Sex 

**Figure 4:**
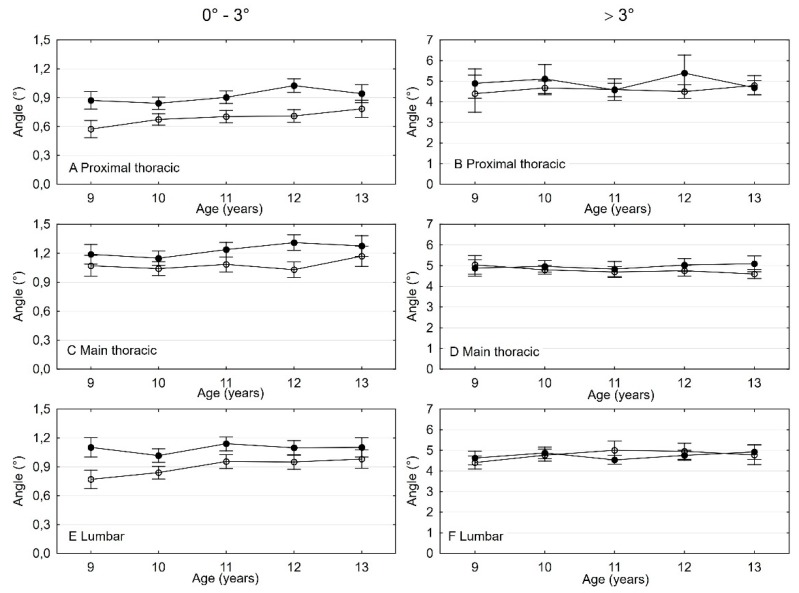
Two-factor analysis of variance to assess differences in ATR value.* * Lines on charts with full bullet points means girls and with empty bullet points boys.

As shown in Figure 4, at all measurement levels in the range 0–3º, girls had higher values of trunk rotation than boys (level 1: F_1-6594_ = 86.94; *p* < 0.0001, level 2: F_1-6121_ = 28.113; *p* < 0.0001, level 3: F_1-6367_ = 50.501; *p* < 0.0001). ATR increased between the age of 9 and 13 years except the second level of measurement where girls and boys did not differ (significant effect of time, level 1: F_4-6594_ = 5.077; *p* = 0.0004, level 3: F_4-6367_ = 4.264; *p* = 0.0019; non-significant effect of time, level 2: F_4-6121_ = 2.225; *p* = 0.0638). ATR increased similarly in girls and boys in all levels (non-significant age x sex inter-reaction, level 1: F_4-6594_ = 1.925; *p* = 0.1034, level 2: F_4-6121_ = 1.537; *p* = 0.1885, level 3: F_4-6367_ = 1.363; *p* = 0.2441). In the range >3º were no changes in ATR between girls and boys (level: F_1-236_ = 2.631; *p* = 0.1061, level 2: F_1-709_ = 2.723; *p* = 0.0993, level 3: F_1-463_ = 0.095; *p* = 0.7578), non-significant effect of time (level 1: F_4-236_ = 0.568; *p* = 0.6863, level 2: F_4-709_ = 0.351; *p* = 0.8434, level 3: F_4-463_ = 0.677; *p* = 0.6083), and non-significant age x sex inter-reaction (level 1: F_4-236_ = 1.053; *p* = 0.3804, level 2: F_4-709_ = 0.643; *p* = 0.6323, level 3: F_4-463_ = 1.377; *p* = 0.2406).

### 3.4. The Difference and Comparison in Body Weight Values Between Age and Sex 

**Figure 5:**
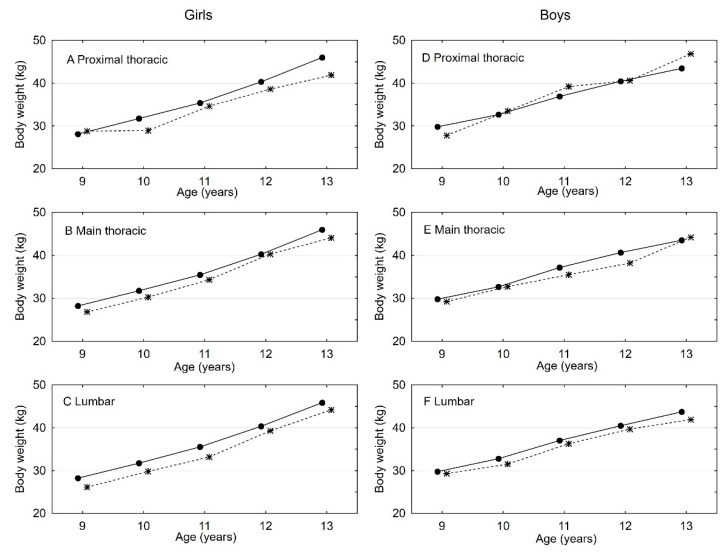
Two-factor analysis of variance to assess differences in ATR value with body weight. * * Section of ATR = 0–3º is presented with solid lines, section of ATR > 3º with dotted lines.

As shown in Figure 5, it is worth mentioning that girls with ATR > 3º at all measuring levels are significantly slimmer than girls with ATR 0–3º (level 1: F_1-3430_ = 4.139; *p* = 0.0420, level 2: F_1-3430_ = 5.67; *p* = 0.0173, level 3: F_1-3430_ = 8.98; *p* = 0.0028). In boys, the differences in average size of body mass over the individual age groups were very similar (level 1: F_1-3430_ = 1.002; *p* = 0.3170, level 2: F_1-3430_ = 1.85; *p* = 0.1736, level 3: F_1-3430_ = 1.95; *p* = 0.1628).

## 4. Discussion 

School scoliosis screening (SSS) using the scoliometer require a value that indicates minimal changes to determine whether the patients need clinical treatment [21]. Coelho et al. [13] showed that it is possible to identify 87% of the patients with IS lateral curvatures greater than 10º Cobb and 100% of the patients with curves greater than 20º Cobb using 5º as the criteria for referral. Bunnell went to a minimum 7º angle of trunk rotation as a criteria for referral to decrease the number of false positives and recommended that children with a lower degree of trunk deformity (4–6º trunk rotation), which can present mild scoliosis, should be rescreened in 4–12 months [20,22]. 

Optimal management of idiopathic scoliosis requires prevention rather than treatment of disease. The presented research using a referral criterion of 5º ATR indicating its potential for screening participants with idiopathic scoliosis, showed that in the group with more than 3º ATR, 30.5% of respondents had a 5º value. In addition, a high percentage of participants with a five-degree value of ATR was also noted on the third measuring level—31.1%.

Our findings indicate trunk asymmetry correlates well with age, gender, and body mass. We found strong correlations of ATR value with age and sex. Over the analyzed material, more girls had a 5º ATR value than boys.

The optimal age screening for scoliosis is still under debate. Characterizing the relationship between different groups of age, we found a significant effect in the group of girls. In girls, the main thoracic and thoraco-lumbar level of measurement showed the significant effect of difference at age 11. Changes at the level of proximal thoracic presented statistically significant differences at the age of 13. Research results indicated the most significant level of measurement. Our findings suggest that girls should be screened for IS on the main thoracic and thoraco-lumbar section at the age of 11. The U.S. Preventive Services Task Force concludes that the current evidence is insufficient to assess the balance of benefits and harms of screening for adolescent idiopathic scoliosis in children and adolescent aged 10 to 18 years [23]. Trunk distortion of scoliosis involves other structures than the spine. Some researchers have suggested that the Cobb angle purely attains to the spinal deformity, while the ATR reflects a truncal and spinal deformity together [24].

Some studies conducted overseas have reported that low weight contributes strongly to the occurrence of scoliosis [25]. Unequal body mass in the girls group, an ATR of 0–3º and ATR > 3º also should be considered. In the entire population of girls with ATR > 3º, body mass was lower than in a group with ATR 0–3º, which means that girls who are slimmer are predisposed to asymmetry of trunk rotation during the developmental stage. Cheung at al. (2003) in their research present that lower BMI means lower muscle mass which increases the risk of scoliosis [26]. Kyoungkyu at al. (2018) indicated that school students aged 10 to 14 years old in underweight and severely underweight groups had significantly higher risks of developing scoliosis, with risk levels that were 1.43 times and 1.45 higher, respectively [27]. As such, maintaining an appropriate weight level is very important for reducing the risk of scoliosis. 

### Strengths and Limitations

This research has some limitations. Respondents of this study were elementary school students applied to five age groups. Therefore, data cannot be extrapolated to other age ranges. Furthermore, there is still no consensus about the age at which school scoliosis screening should be started. The above research attempts to assess that age. In addition, it presents the level of the spine and which examination for IS detection cannot be missed. 

## 5. Conclusions 

Age, sex, and the risk of developing angle of trunk rotation are very closely associated. The main thoracic (level 2) and thoraco-lumbar (level 3) level of measuring the spine appears to be the most differentiating in the diagnosis of scoliosis. Therefore, in practice, researchers should focus mainly on these levels. Study restriction will also reduce the time for examining a child. By using a variety of statistical analyses, our findings emphasize that girls with a lower degree of trunk deformity (4–6º trunk rotation), which can present mild scoliosis and those with a higher degree (7º trunk rotation), have lower body mass than girls within the norm. Our results suggest that among Polish elementary school students, the angle of trunk rotation should be measured in girls on the second and third level until 11 years of age. The first measuring level should be checked two years later. In the group of boys, second level of measurement appears to be diagnostic until 13 years of age. There is still no gold non-invasive standard for preventive scoliosis screening. It will be a good idea to involve a scoliometer measurement into children’s health care observation. The examination is simple and only needs previous short physiotherapy training. In our opinion, it can be done also by school nurses or teachers of physical education. 

## Figures and Tables

**Table 1:** Anthropometric characteristics (*n* = 6850), mean, minimum, maximum, and standard deviation of the participants showing the differences between girls and boys and the *p*-values for Z-test.

Gender	Female (*n* = 3440)			Male (*n* = 3410)			*p*-Value of Difference *
Parameters	Mean	Min–Max	SD	Mean	Min–Max	SD	*p*
Age (years)	10.98	9–13	1.23	10.99	9–13	1.23	0.5991
Mass (kg)	35.93	16–89	10.15	36.66	16.8–90	10.15	**0.0029**
Height (cm)	140.84	114–179	9.91	141.25	114–174.5	9.03	0.0735
BMI	17.84	7.2–38.5	3.32	18.13	11.1–42.0	9.91	**0.0004**
Level 1 (°)	1.08	0–15	1.28	0.82	0–10	1.16	**<00001**
Level 2 (°)	1.67	0–21	1.66	1.41	0–12	1.51	**<0.0001**
Level 3 (°)	1.39	0–10	1.44	1.13	0–13	1.37	**<0.0001**

*p < 0.0001.

**Table 2:** Percentage distribution of children with angle of trunk rotation (ATR) = 5º.

	N (100%)	ATR = 5°	
		N	%
Level 1 ATR > 3°	N = 246	71	28.9
Level 2 ATR > 3°	N = 719	226	31.5
Level 3 ATR > 3°	N = 473	157	33.2
Level 1 ATR ≥ 0°	N = 6850	71	1
Level 2 ATR ≥ 0°	N = 6850	226	3.3
Level 3 ATR ≥ 0°	N = 6850	157	2.3
